# Evidence of Failed Resolution Mechanisms in Arrhythmogenic Inflammation, Fibrosis and Right Heart Disease

**DOI:** 10.3390/biom12050720

**Published:** 2022-05-19

**Authors:** Rim Younes, Charles-Alexandre LeBlanc, Roddy Hiram

**Affiliations:** 1 Montreal Heart Institute (MHI), Montreal, QC H1T 1C8, Canada; rim.younes@icm-mhi.org (R.Y.); leblanc.charles-alexandre@icm-mhi.org (C.-A.L.); 2 Department of Medicine, Faculty of Medicine, Université de Montréal, Montreal, QC H3T 1J4, Canada

**Keywords:** inflammation, fibrosis, resolution, right heart disease, atrial fibrillation

## Abstract

Inflammation is a complex program of active processes characterized by the well-orchestrated succession of an initiation and a resolution phase aiming to promote homeostasis. When the resolution of inflammation fails, the tissue undergoes an unresolved inflammatory status which, if it remains uncontrolled, can lead to chronic inflammatory disorders due to aggravation of structural damages, development of a fibrous area, and loss of function. Various human conditions show a typical unresolved inflammatory profile. Inflammatory diseases include cancer, neurodegenerative disease, asthma, right heart disease, atherosclerosis, myocardial infarction, or atrial fibrillation. New evidence has started to emerge on the role, including pro-resolution involvement of chemical mediators in the acute phase of inflammation. Although flourishing knowledge is available about the role of specialized pro-resolving mediators in neurodegenerative diseases, atherosclerosis, obesity, or hepatic fibrosis, little is known about their efficacy to combat inflammation-associated arrhythmogenic cardiac disorders. It has been shown that resolvins, including RvD1, RvE1, or Mar1, are bioactive mediators of resolution. Resolvins can stop neutrophil activation and infiltration, stimulate monocytes polarization into anti-inflammatory-M2-macrophages, and activate macrophage phagocytosis of inflammation-debris and neutrophils to promote efferocytosis and clearance. This review aims to discuss the paradigm of failed-resolution mechanisms (FRM) potentially promoting arrhythmogenicity in right heart disease-induced inflammatory status.

## 1. Introduction

Cardiac diseases, including atrial fibrillation (AF), the most common form of arrhythmia, are characterized by an unresolved inflammatory status [1,2,3]. In response to cardiac injury, apoptotic cardiomyocytes (CM) contribute to activating the inflammatory status regulated by pro-inflammatory signals released by cardiac cells and recruited inflammatory cells [4]. These events characterize the acute phase of inflammation, aiming to promote wound cleaning and to start the healing process [5]. Resolution-promoting signals are then secreted to stop acute inflammation via the initiation of the resolution phase, allowing the maintenance of homeostasis [6]. Cardiac fibroblasts (FB) are sensitive to circulating and CM-originated inflammatory signals [7]. When resolution is successfully activated, pro-resolution processes promote FB-secreted collagenous material to consolidate the extracellular matrix, compensate for the loss of apoptotic CM, and preserve the mechanical stability of the myocardium to protect the heart from rupture and failure [5,8]. In contrast, myocardial remodeling could become dangerous when the acute inflammatory period is prolonged and when the resolution response fails to occur [5,9]. This can lead to a switch into a persistent inflammatory status instead of resolving the inflammation [5,10].

**Figure 1:**
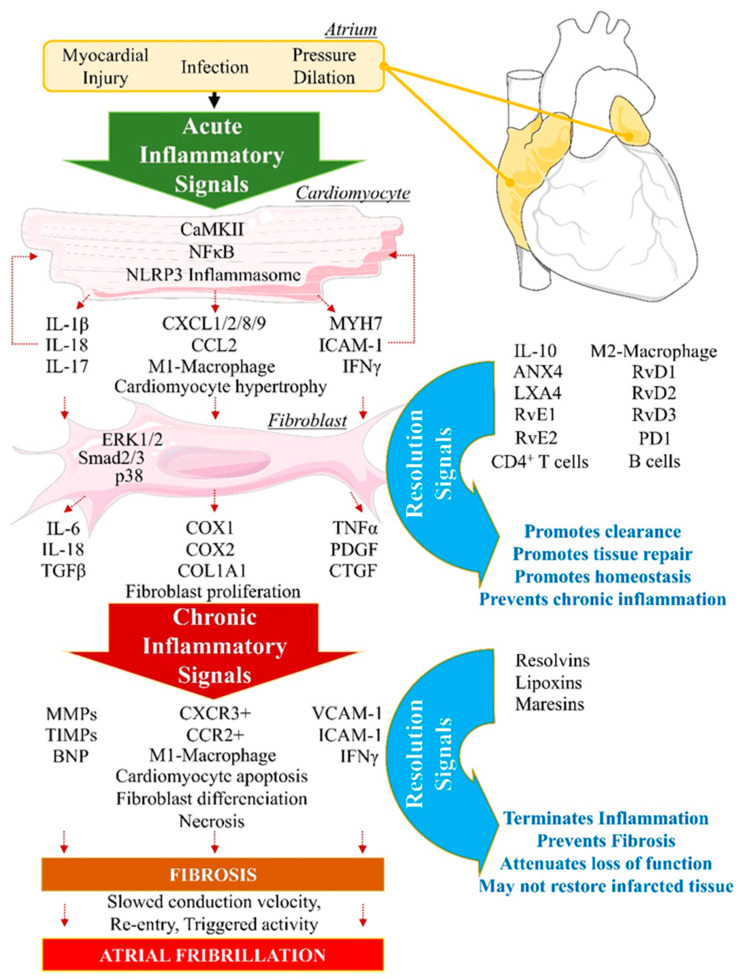
Biomolecular orchestration of cellular events from cardiac insult to resolution opposed to persistent arrhythmogenic inflammation. Longstanding exposure of the atrium to myocardial injuries, infections or chronic pressure and dilation provokes the normal initiation of acute inflammation. In cardiomyocytes (CM), intracellular inflammatory response involves CamKII, NF-kB or NLRP3 inflammasome pathways activation, which contribute to CM deregulation of structural genes (*Myh7*), and secretion of proinflammatory cytokines including interleukins (IL-1β, IL-18) and chemokines (CXCL, CCL), leading to promotion of proinflammatory (M1)-macrophage infiltration. Proinflammatory signals contribute to the activation of cardiac fibroblasts (FB). FB activate additional pro-inflammatory signals (TGFβ, TNFα, PDGF) provoking FB differentiation into myo-FB, aiming to promote repair and wound healing, if the resolution signals are properly activated in response to inflammation initiation. Resolution mediators, including IL-10, LXA4, D- and E-series resolvins, contribute to terminate M1-macrophages infiltration, facilitate anti-inflammatory (M2)-macrophages polarization and phagocytosis, while activating CD4+ T cells and B cells efferocytosis, leading to homeostasis. When Resolution fails to occur, inflammation is perpetuated via FB and myoFB secretion of chronic-inflammation-promoting mediators (MMPs, IFNγ, CXCR3+, M1-macrophages) leading to CM necrosis, and loss of function. Resolution signals can be promoted to limit chronic inflammation-induced damages. If failed resolution mechanisms persist, the myocardium is exposed to the development of fibrosis, slowed conduction velocity, triggered activity, re-entry and increased susceptibility to arrhythmias, including atrial fibrillation.

Chronic inflammatory signals promote fibrotic tissue deposition, constituting a “stiff” layer on the myocardium [11]. Such fibrous zones are non-contractile and electrical insulator areas that disrupt the normal propagation of action potential can cause conduction slowing, refractoriness and AF [12,13] (Figure 1). Pro-resolution therapeutic strategies are poorly described in the field of anti-arrhythmic drug-development and arrhythmia-management.

Among cardiac disorders with an important inflammatory impact, right heart disease (RHD) is a pathological condition in which the right ventricle (RV) suffers from a structural and electrical remodeling that strongly affects cardiac physiological functions [14]. Right heart structure, heart chambers, and the circulatory system are vulnerable to morphological modifications that may result from hypertension-promoting cardiac conditions, including pulmonary artery hypertension (PAH), chronic obstructive pulmonary disease (COPD) or pulmonary embolism [15]. Volume- and pressure-overload conditions associated with structural remodeling negatively impacts the cardiac function, particularly because of the induced inflammatory status, and can potentially result in myocardial fibrosis in response to a chronic rise in blood pressure, myocardial tissue stretching, or myocardial injury [16]. In the RV and the right atrium (RA), electrical remodeling is at the origin of potential tachycardia and arrhythmias, including ventricular fibrillation or/and atrial fibrillation (VF and AF) [17,18]. In response to structural remodeling, pro-inflammatory cytokines, and chemokines such as IL-1β, IL6, IL18, TGF-β, or CXCL1/2 stimulate fibroblasts (FB) differentiation into myofibroblasts (myo-FB) associated with a gradual loss of function in the myocardium [19,20]. Events and conditions promoting the development of cardiac fibrosis in the atrial tissue are associated with arrhythmogenic structural and functional modifications, promoting AF [3,21,22] (Figure 1).

The current article aims to review: (I) the general biochemical paradigm orchestrating the active mechanisms of resolution and the relevance of the concept of failed resolution mechanisms (FRM) in cardiac disorders; (II) the clinical and experimental investigations that tried to understand the role of cardiac FBs in the different phases following cardiac injury, including initiation of inflammation, resolution, chronic inflammatory phase, and arrhythmogenic cardiac remodeling including RHD; and (III) the importance of considering FRM in an understanding and therapeutic management of RHD associated with arrhythmogenic atrial remodeling. We finally discuss the relevance of novel molecular targets that could potentially help to switch chronic inflammation into resolution and homeostasis, in order to prevent cardiac arrhythmias and AF.

## 2. Biomolecular Paradigm of Active Resolution Mechanisms in the Heart

### 2.1. Initiation Phase of Inflammation: Central Regulatory Role of Arachidonic Acid

During the initiation of acute inflammation, phospholipase A2 (PLA2) levels are increased at the site of injury [23]. PLA2 produces arachidonic acid (AA: 5, 8, 11, 14-eicosatetraenoic acid) by hydrolyzation of the sn-2 ester bond of cellular phospholipids [23]. Patients with coronary artery disease show increased levels of lipoprotein-associated PLA2 (Lp-PLA2) [24]. Elevated levels of Lp-PLA2 have been suggested as an important risk factor of cardiovascular diseases [25]. Paradoxically, when Lp-PLA2 hydrolyzes the platelet-activating factor (PAF), its enzymatic activity is associated with anti-inflammatory properties [26]. The underlying mechanisms governing this paradox will be discussed below.

#### 2.1.1. Arachidonic Acid Metabolism by Cytochrome P450

**Figure 2:**
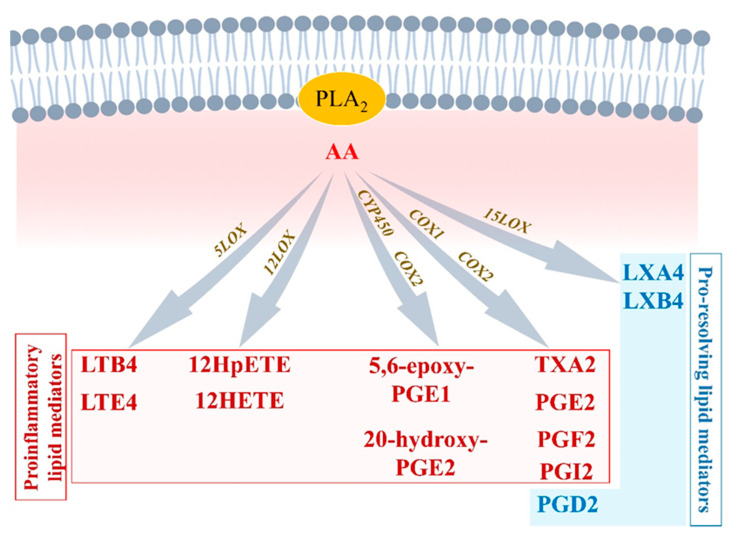
Arachidonic acid-derived lipid mediators. Arachidonic acid (AA) interaction with COX1, COX2, 5LOX, 12LOX, or CYP450 enzymes mainly leads to the production of proinflammatory lipid mediators including leukotrienes, thromboxanes, and prostaglandins. AA interaction with COX1/2 or 15LOX can generate pro-resolution mediators including PDG2, LXA4, and LXB4.

AA is an essential polyunsaturated fatty acid (omega-6 PUFA) that can interact with cytochrome P450 (CYP450) enzymes to undergo monooxygenation or epoxidation and produce hydroxyeicosatetraenoic acids (19- and 20-HETEs) and dihydroxyeicosatrienoic acid (diHETrEs) [27] (Figure 2). These molecules act as hormone-like autocrine and paracrine agents to promote vasoconstriction, vascular permeability, polymorphonuclear leukocytes (PMN), and proinflammatory (M1)-macrophages chemotaxis, and proinflammatory signaling [28].

#### 2.1.2. Arachidonic Acid Metabolism by COX1 and COX2

AA can directly interact with COX1 and COX2 to produce prostaglandin H2 (PGH2), an intermediate metabolite that is converted into bioactive proinflammatory lipid mediators such as thromboxane A_2_ (TXA_2_), prostaglandin A_2_ (PGA_2_), PGB_2_, PGE_2_, and PGI_2_ (Figure 2). These AA metabolites have been shown to be elevated in various cardiovascular conditions, including hypertension, atherosclerosis, vasculopathy, and myocardial infarction [29]. AA-derived lipids mediate vasoconstriction, increase vascular permeability, and stimulate expression of proinflammatory chemokines (complement component (C): C3b, C5a; chemokine C-X-C motif ligand 1 (CXCL1), CXCL2, CXCL8) and interleukins (IL1β, IL6, IL8, IL18, tumor necrosis factor alpha (TNFα)) to promote polymorphonuclear leukocytes (PMN) and proinflammatory-(M1)-macrophages chemotaxis and adhesion, by increasing expression of intercellular adhesion molecule 1 (ICAM1), vascular cell adhesion molecule 1 (VCAM1), and e-selectin (SELE), which act on endothelial cells to promote the adherence of neutrophils to the blood vessel wall [29,30]. These inflammatory biomarkers have been described to promote the development and progression of cardiovascular diseases including cardiac arrhythmias and AF [11,12,22].

#### 2.1.3. Arachidonic Acid Metabolism by 5-LOX

AA can also interact with 5-LOX to produce 5-Hydroperoxyeicosatetraenoic acid (5-HpETE), which promotes vasoconstriction. 5-HpETE can be metabolized either by leukotriene (LT) C-synthase to produce LTC4, LTD4, and LTE4, or by LTA-hydrolase to produce LTB4 via LTA4, which are all leukotrienes playing proinflammatory properties by amplifying PMN and M1 macrophages influx in the injured tissue [31] (Figure 2). HETEs have been shown to activate the nuclear factor kappa-light-chain-enhancer of activated B cells (NFκB) signaling to promote abnormal CM hypertrophy [32].

Evidence shows that AA-derived metabolites’ receptors are expressed on most cardiac cells including CM and FB [33]. In CM, inflammation signaling promotes NFκB activity and the assembling of the NACHT, LRR, and PYD domains containing protein 3 (NLRP3) inflammasome, leading to secretion of IL-1β and increased inflammatory status [34]. Patients with AF have shown increased expression of IL-1β and NLRP3 inflammasome components [35]. Normal initiation of inflammation must be followed by bio-molecularly orchestrated cellular processes aiming to terminate the inflammatory state and promote resolution [36]. In this purpose, lipid-mediator (LM) class switching is a key event that could be defined as a transition phase between the end of inflammation-initiation and the beginning of resolution [37].

### 2.2. Lipid-Mediator Class Switching: Transition from Pro-Inflammatory to Pro-Resolution Signals

During the initiation phase of inflammation, neutrophils have an intense apoptotic and phagocytic activity [38]. This activates intracellular accumulation and extracellular secretion of 12/15-LOX in the damaged tissue. This accumulation of 12/15-LOX activates lipid-mediator-class switching from proinflammatory to pro-resolution mediators [39]. AA is then enzymatically metabolized by 12/15-LOX into lipoxins, including lipoxin (LX) A4 and LXB4 (Figure 2). LXA4 activates its transmembrane specific-receptor lipoxin A4 receptor or formyl peptide receptor 2 (ALX/FPR2) expressed on PMN and macrophages to limit further leukocyte trafficking, stimulate monocyte recruitment, promote anti-inflammatory (M2)-macrophage polarization, and activate phagocytosis and elimination of debris [39]. LXA4 has been shown to be significantly decreased in patients with chronic heart failure [40]. Recent studies have shown that LXA4 attenuates myocarditis by inhibiting NFκB and PI3K/Akt signaling pathways. 15-epi-LXA4 promotes initiation of resolution after myocardial infarction [41,42]. This activity of LXA4 suggests that, in arrhythmogenic conditions, anti-resolution signals promote the diminution of LXA4 production or/and LXA4-associated activity and signaling [42]. LXA4 could be an interesting candidate in the prevention of inflammation-induced substrate of arrhythmias, including AF.

### 2.3. Resolution of Inflammation: SPMs-Mediated Efferocytosis and Homeostasis

**Figure 3:**
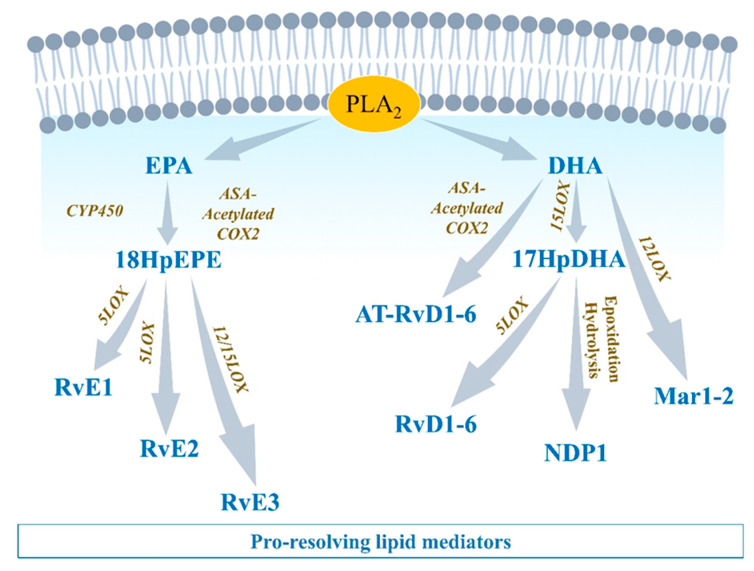
Eicosapentaenoic acid-, and docosahexaenoic acid-derived lipid mediators Eicosapentaenoic acid (EPA) and docosahexaenoic acid (DHA) compete with AA in interacting with COX1/2, 5LOX, 12LOX, and 15LOX. Lipids produced from EPA and DHA metabolism include E-series resolvins (RvE1-3) and D-series resolvins (RvD1-6), respectively, which are involved in pro-resolution mechanisms.

Along with AA, other essential n3PUFAs are delivered with edema fluids at the site of injury. Among them, eicosapentaenoic acid (EPA) and docosahexaenoic acid (DHA) compete with AA to be enzymatically metabolized by either CYP450, 5LOX, 12LOX, 15LOX, or aspirin-acetylated COX2 [43]. Knowing that AA-derived metabolites are crucial in the initiation of normal inflammation, and that EPA and DHA products are important in resolution, it is understandable that optimal healthy conditions must promote a fair balance between AA, EPA, and DHA concentrations. Hence, in opposition to what has long been thought, it is not recommended to completely annihilate AA-derived metabolites (i.e., by using COX inhibitors) to guaranty homeostasis [44] (Figure 2 and Figure 3).

#### 2.3.1. EPA-Derived Specialized Pro-Resolving Mediators

EPA is metabolized by CYP450 or aspirin-[ASA]-acetylated COX-2 into 18-HpEPE (18R-hydroperoxy-5Z, 8Z, 11Z, 14Z, 16E-eicosapentaenoic acid), which itself can interact with either 5LOX to produce E-series resolvins (Rvs), RvE1 and RvE2 or 15LOX to produce RvE3. E-series Rvs activate specific receptors such as chemokine-like receptor 1 (CMKLR1), also known as chemerin receptor 23 (ChemR23) (receptor of RvE1), or antagonize proinflammatory leukotriene receptors, such as leukotriene B4 receptor 1 (BLT1), expressed on PMN cell membrane, to stop the expression of chemoattractants, limit neutrophils adhesion/infiltration, and promote phagocytosis of apoptotic neutrophils and efferocytosis [45] (Figure 3).

#### 2.3.2. DHA-Derived Specialized Pro-Resolving Mediators

**Figure 4:**
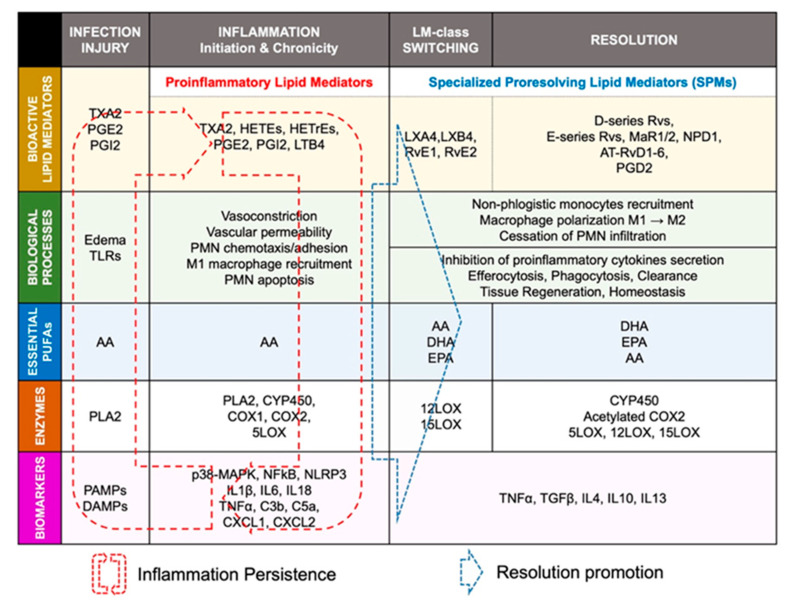
Categorization of inflammation- and resolution-promoting agents. In response to pathogens or injury stimuli (PAMPs, DAMPs), phospholipase A2 (PLA2)-induced biosynthesis of arachidonic acid (AA) leads to the production of proinflammatory lipid mediators including thromboxanes, leukotrienes, and prostaglandins. Such events mark the initiation phase of acute inflammation characterized by PMN chemotaxis, pro-inflammatory-[M1]-macrophages recruitment, and enhanced proinflammatory signals (NLRP3 inflammasome, NF-κB, IL-1β, CXCL1/2). Phagocytic M1-macrophages release 12/15 LOX, which promotes the activation of lipid-mediators (LM) class switching, where AA, docosahexaenoic acid (DHA), and eicosapentaenoic acid (EPA) interact with 12LOX and 15LOX enzymes to be metabolized into specialized pro-resolving mediators (SPMs) including PGD2 from AA, D-series resolvins from DHA, or E-series resolvins from EPA. SPMs promote anti-inflammatory (M2)-macrophage recruitment, inhibition of proinflammatory cytokines’ secretion, termination of inflammation, and regeneration of optimal functions. When the resolution mechanisms fail to occur, inflammation is perpetuated. Inflammatory mediators are overexpressed, leading to persistence of cellular damages, necrosis, fibrosis, loss of function and cardiac vulnerability to arrhythmias, and heart failure. Strategies promoting the augmentation of pro-resolution mechanisms can potentially limit, or eventually reverse, some chronic-inflammation-induced cardiac disorders.

DHA can be metabolized by 12LOX to produce maresins (MaR1-2), or 15LOX to produce D-series resolvins (RvD1-6) and neuroprotectin D1 (NPD1) [46]. DHA interaction with aspirin-acetylated COX2 results in aspirin-triggered resolvins (AT-RvD1-6), which have been described to have similar properties as their classic homologs of the D-series Rvs [47] (Figure 3). D-series resolvins activate specific receptors such as ALX/FPR2 (receptor of RvD1), G-protein-coupled receptor 32 (GPR32: receptor of RvD1 and RvD3), and GPR18 (receptor of RvD2) that are expressed on vascular endothelial cells [37]. The activation of these signals promotes eNOS and P-ERK1/2 signaling, vascular permeability to non-phlogistic monocytes, cessation of PMN infiltration, macrophage polarization into M2-macrophages, M2-macrophages phagocytosis of cellular debris, and maintenance of homeostasis [37,45] (Figure 4).

#### 2.3.3. Arachidonic Acid-Derived Specialized Pro-Resolving Mediators

The metabolism of AA by COX does not only generate proinflammatory components. PGD2 has been shown to play an important role in resolution of inflammation [48]. PGD2 interacts with prostaglandin-D2-receptor 1 and 2 (DP1/2) expressed on T helper type (Th2) cells and dendritic cells that are involved in efferocytosis, phagocytosis, and clearance, to promote elimination of debris and pathogens, and induce complete resolution [48]. The interaction of AA with CYP450 can lead to production of epoxyeicosatrienoic acids (EETs) that are converted by soluble epoxide hydrolase (sEH) into dihydroxyeicosatrienoic acids (DiHETrEs). Although DiHETrEs are toxic, it has been shown that EETs mostly play a beneficial role by promoting vasorelaxation, and cardioprotective effects [49] (Figure 2 and Figure 4).

### 2.4. ‘Failed Resolution Mechanisms’ in the Development of Chronic Inflammation and Heart Diseases

Lipid mediator (LM) production and signaling are fundamental in the regulation of the normal process of acute inflammation from its initiation to its resolution [46]. When the cardiac tissue undergoes a chronic inflammatory status, the crucial phase of LM class switching, which promotes the end of PMN infiltration and the activation of efferocytosis, may have failed to promote the shift of the cellular and lipidic accumulation from proinflammatory to pro-resolution mediators in the injured tissue [39]. Pathologic failure in the production of 12/15 LOX by immune cells including eosinophils, PMN, lymphocytes, and macrophages, leads to a lack of metabolization of AA into lipoxins (LXA4, LXB4) [38]. Lipoxins are essential to activate the cessation of neutrophil recruitment and the infiltration of non-phlogistic monocytes in the site of injury, which is the first step of resolution [38,39]. Moreover, lack of 12/15LOX prevents the production of D-series Rvs from DHA and RvE3 from EPA [38,50] (Figure 3). This may contribute to an annihilate resolution. Then, more proinflammatory LMs (Prostaglandins, leukotrienes) are produced from AA enzymatic interactions with the other enzymes available (COX2, CYP450, 5LOX) [50]. Abnormal accumulation of proinflammatory signaling promotes the prolongation of the initiation phase, characterized by the persistence of the external, cellular, and molecular signs of inflammation. This chronic inflammatory status leads to the development of fibrosis and loss of function [40,50]. If the local production of 12/15LOX is restored, or if the bioavailability of resolvins and lipoxins is increased (from diet or endogenous biosynthesis) at the site of injury, the tissue may enter the resolution phase via termination of proinflammatory signals, reduction of fibrosis, wound healing, and restoration of homeostasis [39,51,52] (Figure 1 and Figure 4).

The detailed biomolecular characterization of inflammation–resolution remains partially understood in cardiac conditions. Moreover, each cardiac disease may display specific biomarkers involved in the incidence of the disorder. Although recent studies suggest a role of SPMs in ischemia-reperfusion [42,53] and pulmonary arterial hypertension-induced right atrial arrhythmogenic substrate [54], further fundamental research and clinical studies are required to assess whether resolution-promoting strategies and cytokine therapies could be an efficient approach to prevent and treat cardiac diseases, including hypertrophic cardiomyopathy, dilated cardiomyopathy, valvopathy, myocardial infarction, or arrhythmias.

## 3. Description of ‘Failed Resolution Mechanisms’ in Cardiac Arrhythmogenic Remodeling

In cardiac arrhythmias, the available knowledge about the pathophysiology of ventricular or atrial fibrillation and their risk factors suggests that not only inflammation signals, but also specific breakdown in the active resolution machinery that we define as “failed resolution mechanisms” (FRM), may play a key role in the occurrence and development of the arrhythmogenic substrate [20,22,55].

Various systemic conditions reported to affect the heart are responsible for the circulating inflammatory agents involved in the development, aggravation, and persistence of cardiac diseases including arrhythmias [22]. Among the disorders that have a clinically relevant impact on the incidence of cardiac inflammation and arrhythmias, are the following: obesity, diabetes, obstructive lung diseases, gastrointestinal disorders, chronic kidney disease, liver cirrhosis, or neurodegenerative diseases [15,56,57,58,59,60].

Although inflammatory cytokines seem involved in arrhythmogenic cardiac disorders, pharmacological strategies targeting inflammatory and resolution systems are not currently standardized as antiarrhythmic medications [56]. Mounting evidence suggests that such therapeutic strategies may represent a promising avenue to explore in the clinical management of cardiac arrhythmias [13,61].

### 3.1. FRM Associated with Cardiac Electrical Conduction Abnormalities

**Figure 5:**
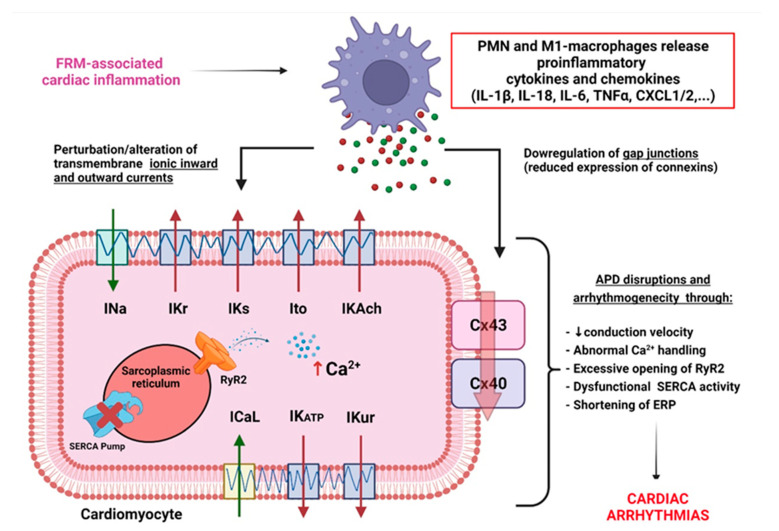
FRM-induced arrhythmogenic channelopathy in cardiac myocytes. In the myocardium, failed resolution mechanisms are associated with increased infiltration of polymophonuclear leukocytes (PMN), enhanced M1-macrophages phagocytic activity and augmentation of the secretion of proinflammatory cytokines. Persistence of proinflammatory signals provokes deregulation of gene coding for key ion-channels and connexins involved in the establishment of cardiac action potential (AP) and conduction velocity. Inflammation-associated channelopathy induces: abnormal calcium-(Ca^2+^)-handling, effective refractory period (ERP) shortening, reduced AP duration (APD), slowed conduction, vulnerability to re-entry, and triggered activity leading to increased risk of cardiac arrhythmias and fibrillation.

Inflammatory cytokines have been reported to directly affect cardiac remodeling by promoting electrical changes early after the initiation of inflammation [22,61]. If unresolved, inflammatory cytokines’ release promotes an alteration of the CM transmembrane activity of inward depolarizing cation currents (including sodium INa, and calcium current ICaL) [61,62]; and the perturbation of outward depolarizing potassium currents, including IK1, Ito, IKr, IKs, IKACh, IKATP, and IKur [61,63]. Dysfunction of these ion channels is associated with abnormal action potential duration (APD), leading to myocardial refractoriness, promoting arrhythmogenicity [64]. In addition to electrical remodeling, inflammation signals promote gap-junctions’ downregulation, leading to lateralization and decreased expression of connexin (Cx) 40 and Cx43 [65]. These inflammation-induced channelopathies contribute to a slowed conduction velocity, promoting re-entry [53,54,55,60,61,62,63,64,65]. FRM-mediated chronic inflammation and CM remodeling promote abnormal intracellular Ca^2+^-handling due to a pathological increase in Ca^2+^-loading, RyR2-opening, and/or dysfunctional sarco-endoplasmic reticulum Ca^2+^-ATPase (SERCA) activity, contributing to ectopy and triggered activity [66,67,68]. It is suspected that the prevention of FRM and the control of inflammation and fibrosis may have cardioprotective effects in order to preserve normal myocardial conduction properties and limit arrhythmia occurrence [54,68] (Figure 5).

### 3.2. FRM Associated with Cardiac ECM’s Arrhythmogenic Structural Remodeling

**Figure 6:**
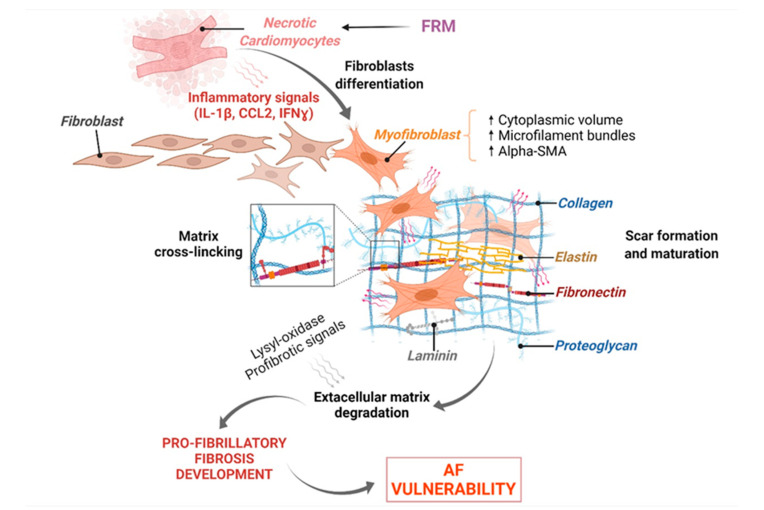
Cardiomyocytes and Fibroblasts’ orchestration of arrhythmogenic extracellular matrix degradation. During acute inflammation, failure in the occurrence of resolution mechanisms (FRM) promotes exacerbation of inflammatory signals generated from apoptotic cardiac cells and activated cardiac FB, leading to an aggravation of myocardial damages. The extracellular matrix (ECM) is intensively remodeled and degraded, promoting a persistence of fibrosis, scar formation, abnormal cardiac function, and increased risk of arrhythmias, including AF.

The extracellular matrix (ECM) is a complex network consisting of glycoproteins, proteoglycans, and glycoaminoglycans including fibers, collagen, fibronectin, laminin, and elastin, surrounding cardiac cells to provide structural support and strength [69]. FB perform important secretory activity to maintain the integrity and regulation of the ECM [70]. After the initiation of inflammation, resident FB are activated and recruited to the site of injury to initiate reparative processes [69,70]. Inhibitors of metalloproteinases are activated in order to limit acute inflammation and protect newly synthetized ECM from degradation [71]. When resolution and termination of inflammation fail, FRM are promoted by persistent inflammatory signaling, triggering FB differentiation into myo-FB by acquiring various phenotypic changes, including a higher cytoplasmic volume, increased microfilament bundles, and upregulated αSMA filament expression [72]. Myo-FB can secrete further ECM components, contributing to a build-up of the myocardium structure and compensating disease-induced CM necrosis. In this context, myo-FB are also able to provide contractile force, stiffening of the ECM and expansion of the fibrotic area [73]. At the later stage of cardiac remodeling, secreted collagen is subjected to cross linking to consolidate the scar [74]. When healing processes fail to promote neither regeneration of the tissue nor repairment, chronicity of inflammation and FRM lead to chronic wound formation characterized by upregulation in the expression of lysyl-oxidase and pro-fibrotic signaling responsible for the maturation of the scars associated with outrageous degradation of the ECM [75,76] (Figure 6).

Chronic inflammation-induced loss of myocardial thickness is associated with cardiac loss of function and aggravation of cardiac disease [77]. FRM-associated degradation of ECM and CM-death is a major challenge in cardiac disease management [78]. It is unclear whether specialized pro-resolving mediators (SPM) could regenerate wounded myocardium. Further studies investigating the impact of resolvins after irreversible scar formation in ischemic cardiac disorders are required to assess their potential regenerative effects and efficiency.

### 3.3. FRM Associated with Abnormal Cardiac Fibroblasts’ Remodeling and Atrial Fibrosis

Cardiac FB are sensitive to cardiac immune response to injury via close interactions with activated inflammatory cells and the tightly regulated healing process initiated to limit tissue damage [74]. FB are involved in the activation of reparative processes that are essential to preserve the proper structure and function of the heart [71,74]. Longstanding and uncontrolled inflammation can lead to FRM, cardiac remodeling, congestive heart failure, cardiac dysfunction, arrhythmia, and sudden death [54,77].

#### 3.3.1. Fibroblast Response to Inflammation Initiation

**Figure 7:**
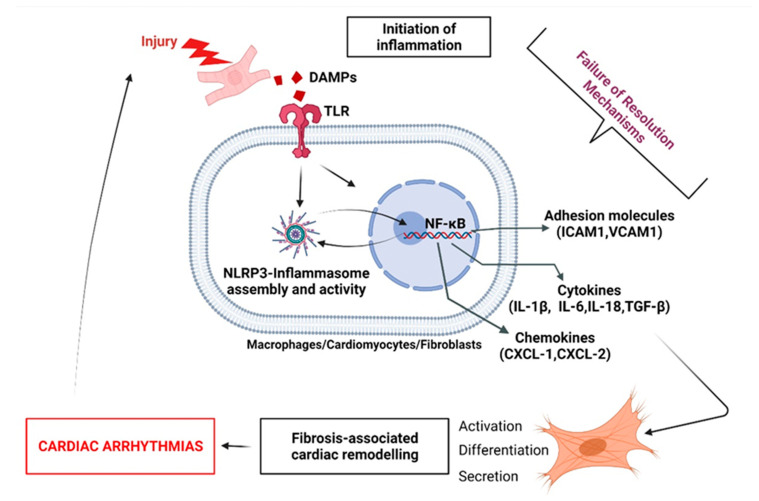
The central role of unresolved fibroblasts-associated inflammatory status in the development of arrhythmogenic cardiac fibrosis. During acute inflammation, the initiation of inflammation is normally followed by a resolution, in order to promote homeostasis. Failure in the activation of pro-resolving signals contributes to the exacerbation of cardiac cells’ release of proinflammatory stimuli. FB are highly sensitive to inflammatory agents. When activated, cardiac FB participate in increasing the production of inflammatory compounds, leading to the development of fibrosis, myocardial dysfunction and arrhythmogenicity.

Injury-induced degradation of ECM generates damage-associated molecular patterns (DAMPs) [79]. Heart injury disrupts CM’s cellular membrane and leads to the release of inflammatory cytokines [79]. Binding of DAMPs to their specific pattern recognition receptors (PRR) present on leukocytes, macrophages, endothelial cells, and resident FB initiates the innate immune response by the activation of a cascade of intracellular proteins, which culminate in the activation of transcription factors such as NF-κB [80]. This molecule is then able to translocate to the nucleus in order to initiate the transcription of genes involved in the inflammation and immune response, such as cytokines (*Il-6*, *Il-18*), chemokines (*Cxcl-1*, *Cxcl-2*), and adhesion molecules (*Icam-1*, *Vcam-1*) to induce the recruitment, activation, differentiation of inflammatory cells, and upregulate the FB expression of proteases to degrade dead cellular debris [81]. If perpetuated, these changes contribute to the differentiation, proliferation, and migration of FB, leading to the development of fibrosis, contributing to loss of cardiac function and arrhythmogenicity [13,22,81] (Figure 7).

#### 3.3.2. FB-Induced Expression of Key FRM-Promoting Biomarkers

NLRP3 inflammasome. Chronic inflammation is suspected to be responsible for the development and progression of various cardiac diseases, including coronary arterial disease (CAD), myocardial infarction (MI), valvulopathy, and cardiac arrhythmias [82,83]. Mounting evidence suggests that the NLRP3 inflammasome plays a central role in modulating chronic inflammation and aggravation of heart disease progression [35,84,85]. In patients with acute myocarditis, the initiation of inflammation is associated with activation of the inflammasome in PMN, FB, and CM, correlating with the severity of heart failure [86]. In myocardial ischemia, the inflammasome aggravates tissue injury and promotes cardiac failure, while an absence or inhibition of the NLRP3 inflammasome leads to improvements in cardiac function in preclinical studies [87]. In the CANTOS trial, inhibition of IL-1β maturation was efficacious in secondary prevention for cardiovascular events in patients with previous MI [88]. Genetic deficiency of NLRP3 was associated with reduced expression of proinflammatory cytokines (TNFα, IL1β, IL6), reduced secretion of proinflammatory PGE2 and LTB4, and increased expression of LXA4 and LXB4, which suggests that the presence of NLRP3 may negatively influence the LM-class switching and promote FRM during inflammation progression [89] (Figure 1, Figure 4 and Figure 7).

Interleukin-1β. IL-1β secreted by leukocytes plays an important role in the activation of inflammatory and fibrogenic pathways during the healing process [90]. IL-1β is a contributor to the pathogenesis of cardiac remodeling through the induction of inflammatory mediators’ synthesis by activated leukocytes [91]. In a study performed on IL-1R-/- knock-out mice (not expressing IL-1β receptor) subjected to coronary occlusion/reperfusion, the immunohistochemical staining of the infarct area with anti-alpha-smooth muscle actin (αSMA) antibodies, anti-macrophages, and anti-neutrophils showed a reduced quantity of infiltrating immune cells and myo-FB [92]. Moreover, the authors observed that animals not expressing an IL-1β receptor had lower levels of cytokines and chemokines secreted compared to wild-type animals [92]. Furthermore, it has been shown that administration of an anti-IL-1β neutralizing antibody, in the acute phase of non-reperfused murine MI, resulted in reduced collagen accumulation in the scar and attenuated adverse remodeling [92]. Finally, IL-1R-/- mice had lower FB-induced secretion of metalloproteinases (MMP-2, MMP-3) [92]. These data suggest that IL-1β is a crucial agent in FRM and fibrosis development—a deleterious event to combat in arrhythmia prevention (Figure 7).

Interleukine-6. In myocardial injury, IL-6 is considered as key regulator of cardiac FB differentiation and FB-induced release of inflammatory factors implicated in amplification of the inflammatory response and tissue remodeling [93] (Figure 1 and Figure 7). In affected areas, when IL-6 binds to its specific receptor (IL-6R), it activates Hyaluronan (HA) synthase (HSA1, HAS2), which is responsible for the formation of an HA-rich environment that provides strength, lubrication, and hydration within the ECM, while regulating FB motility, proliferation, and differentiation [94,95]. In mice subjected to MI induced by LAD surgery (ligation of the left anterior descending coronary artery), antibodies against IL-6 inhibited the expression of HAS1 and HAS2 [96]. Moreover, it has been shown that the presence of αSMA-positive cells in the border zone of the infarct was markedly reduced in mice pretreated with blocking IL-6 antibodies [96]. IL-6 binding to glycoprotein (GP130) on the surface of FBs result in a downstream phosphorylation of STAT3 (signal transducer and activator of transcription 3) that moves to the nucleus and activates the transcription of HA-synthase in order to initiate the formation of HA. HA binding to its receptor CD44 on the FB surface contributes to the release of proinflammatory molecules such as chemokine ligand CCL5 and monocyte chemoattractant protein 1 (MCP1: also known as CCL2) and promotes myo-FB phenotype—an FRM, pro-fibrosis, and pro-arrhythmogenic phenomenon [96].

Transforming growth factor-bêta. TGF-β is expressed by macrophages and cardiac FB [97]. TGF-β binds to its receptors TBRI and TBRII to initiate non-canonical pathways by the activation of p38 kinase in the cell, which in turn can activate the serum response factor (SRF) in the nucleus, to start the transcription and upregulation of TRPC6 (the transient receptor potential cation channel, subfamily C, member 6) [98]. TRPC6, located on the surface of the cell, facilitates calcium (Ca^2+^) entry, leading to the activation of calcineurin (CnA) that enhances the myofibroblastic phenotype conversion by NFAT (nuclear factor of activated T-cells) [99]. Studies have demonstrated that mice infected with recombinant adenovirus expressing TRPC6, have shown increased levels of αSMA, augmented activation of FB, and enhanced expression of fibronectin (FN) domain ED-A [73]. In addition, TRPC6 knock-out in a murine model of MI mice showed a lower count of FB conversion into myo-FB; however, there was a higher rate of mortality due to ventricular rupture because the scar formed was smaller compared with wild-type mice [73]. These data suggest that events promoting TGF-β-induced FB differentiation into myo-FB contribute to FRM and promote the development of arrhythmogenic fibrosis [73] (Figure 7).

The beneficial impact of inhibiting some of the above-described FRM promoters is currently being tested in clinical trials using drugs specifically directed against NLRP3 inflammasome or the IL1 proinflammatory interleukins’ family, including IL1β. Existing potential medications with promising beneficial effects to tackle NLRP3 inflammasome, IL1β or IL8, include Canakinumab, Colchicine, Belnacasan, Pirfenidone, Tranilast, Dapansutrile, Inzomelid, Somalix, or SGLT2 inhibitors [100,101,102,103,104,105,106,107]. Although these molecules show interest in various pathologies, including pulmonary fibrosis, SARS-CoV2, or Parkinson’s disease, their relevance in cardiology remains an open avenue to explore [101,107] (Table 1).

## 4. Arrhythmogenic FRM in the Context of Right Heart Disease

### 4.1. Generalities on RHD-Induced Arrhythmogenicity

Right ventricular (RV) hypertrophy often leads to substantial structural, functional, and electrophysiological changes in the myocardium, contributing to the development of arrhythmogenic substrate and the occurrence of ventricular and/or supraventricular arrhythmias [14,17]. When not monitored, RHD associated with electrophysiological and hemodynamic pathologies could lead to heart failure, stroke, or sudden death [14,15,16]. PAH, COPD, obesity, and obstructive sleep apnea (OSA) are conditions inducing RV hypertrophy, while also elevating the risk of AF [17,108,109,110,111]. It is important to notice that other pulmonary hypertension (PH)-related conditions that are not directly responsible for right-sided remodeling may contribute to chronic exposure of the right-heart to circulating inflammatory signals [108,109,110,111,112]. Left heart diseases (LHD) related to PH (including: PH owing to heart failure with preserved LVEF, PH owing to heart failure with reduced LVEF, valvular heart disease, and congenital or acquired cardiovascular conditions leading to post-capillary PH) are characterized by an increased risk of LA arrhythmogenic remodeling and inflammation [87,108,109,110,111,112,113,114,115,116,117,118]. Moreover, PH-related hypoxia (including developmental lung disorders, or restrictive lung disease) also contribute to cardiopulmonary inflammation and structural changes that may participate in atrial arrhythmogenic substrate [88,108,109,110,111,112,113,114,115,116,117,118]. Indeed, the chronic and deleterious rise in pressure and volume in the RV can induce tricuspid annulus plane deformation, and tricuspid regurgitation, leading to direct structural and functional effects on the right and the left atrium (RA and LA) [15,16,111]. Longstanding exposure to elevated pressure and volume leads to RA and LA tissular stretch and dilation [108,109,110,111]. Prolonged pressure/volume overload activates inflammatory stimuli produced in response to right and left atrial structural remodeling [100]. Thus, the RHD-induced FRM negatively affect the RA and also have potential arrhythmogenic structural, functional, and electrical conduction consequences that are observable on the LA [113]. In response to myocardial remodeling and inflammation, atrial FB play a crucial role in RHD-induced atrial remodeling, following the above-described mechanisms in Section 3.3 [114]. Myo-FB differentiation contribute to the formation of atrial fibrotic tissue. Fibrosis-induced atrial remodeling includes perturbation of electrical circuits, leading to an increased risk of AF [114,115] (Figure 1, Figure 5, Figure 6 and Figure 7).

### 4.2. Resolution-Promoting Strategies in Monocrotaline and Sugen-Hypoxia Models of RHD and FRM Associated with Cardiac Arrhythmias

Conditions affecting the right heart have the potential to trigger the chronic development of atrial fibrosis and AF [115]. Various experimental models have been using animals to better understand the underlying mechanisms of AF. Respectively, the choice of studying AF in rodents, dogs, pigs, ewes, or horses have specific perks, advantages, and limitations [116]. RHD-induced cardiac arrhythmias have mostly been studied using models of provoked pulmonary embolism or pulmonary artery occlusion [114,117,118,119]. Technically, different approaches can be utilized to mimic or experimentally re-create pulmonary obstruction and sustained pressure on overload-induced RV failure [120]. Among the well described methods, we found monocrotaline (MCT) [121], chronic hypoxia chambers [122], sugen-induced hypoxia (SuHx) [123], pulmonary artery banding [124,125], or chronic thromboembolic pulmonary hypertension [126].

The available knowledge about FRM-induced arrhythmogenicity is limited in RHD-induced models of myocardial remodeling. Here we report on some very novel studies that have specifically explored the effect of resolution-promoting molecules in animal models of arrhythmia only in the context of RHD. These investigations were performed in rodent models of RHD induced by MCT and SuHx.

Monocrotaline. Monocrotaline (MCT) injections have been widely used to induce pulmonary arterial hypertension and right-sided cardiac hypertrophy and dilation in rats [110]. The ventricular arrhythmogenic aspects of MCT-induced RV hypertrophy has been described in rats [118]. Recently, it has been shown that specialized pro-resolution treatment with RvD1 can attenuate atrial fibrous content and reduce AF inducibility in rats with MCT-induced RHD and atrial dilation [114]. In 2022, Tianyou Qin and collaborators have shown that administration of Dapaglifozin, a sodium-glucose cotransporter 2 (SGLT2) inhibitor, significantly decreased ventricular fibrosis and attenuated NFκB activity while preventing ventricular arrhythmias and VF vulnerability [127] (Table 1). These investigations consolidate the idea that atrial and ventricular arrhythmias associated with RHD could be attenuated via the prevention of FRM. More studies are required to evaluate the impact of pro-resolution biomarkers promoting anti-arrhythmogenic effects in RHD.

Sugen Hypoxia. Sugen (SU5416), an antagonist of vascular endothelial growth factor receptor 2 (VEGFR2), is used to induce pulmonary hypertension and RHD in rodents [128]. Sugen hypoxia (SuHx) has been shown to increase VF inducibility in rats [119]. Treatment with relaxin (RLX), a heterodimeric polypeptide hormone member of the insulin-like superfamily, prevented ventricular fibrosis and VF vulnerability in SuHx-induced RHD-rats [119]. RLX also prevented atrial fibrosis and AF in a rat model of aging and spontaneous hypertension [129,130]. Anti-arrhythmogenic effects of RLX treatment was associated with decreased expression of TGF-β, matrix metalloproteases 2 and 9 (MMP2 and MMP9), and collagenases 1 and 3 (COLI, COLIII) [119,129,130], suggesting that FRM occurring in cardiac hypertensive disorders could be targeted in the management of cardiac arrhythmias (Table 2).

## 5. Conclusions

Chronic inflammation is a consequence of failure in the inflammatory-response machinery to switch from the initiation phase of inflammation into the active resolution phase mediated by SPMs. AA, DHA, and EPA-derived SPMs directly target efferocytosis promotors to prevent FRM and persistent inflammatory status. Clinical evidence demonstrating decreased plasmatic levels of SPMs in patients with chronic heart failure consolidate the concept of ‘Failed Resolution Mechanisms’ in progressive cardiac diseases, including RHD and AF. SPMs are potential strong therapeutic candidates that are able to promote resolution in inflammation-associated arrhythmogenic cardiac disorders.

## 6. Highlights

- Initiation of inflammation is required to combat cardiac insults.
- Arrhythmogenic events may include inhibition of bio-molecularly active lipid-mediator class-switching and resolution.
- Future therapeutic strategies targeting cardiac inflammation must consider the complex equation of not inhibiting the required initiation-processes of inflammation while promoting resolution mechanisms.

## Figures and Tables

**Table 1:** A list of potential medications targeting key FRM-promoting molecules, currently tested in clinical trials. Available drugs are evaluated in ongoing clinical trials to determine their effects to inhibit NLRP3 inflammasome, IL1B, or IL18 in various conditions associated with an unresolved inflammatory status.

Medication	Role on IL1β or/and NLRP3 Inflammasome		Ongoing Clinical Trials	
	Mode of Action	References	Clinical Trial Theme	Clinical Trial ID
Belnacasan	CASP-1 specific inhibitor, Prevents Il-1 β release	[102]	SARS-CoV-2	NCT05164120
Canakinumab	Inhibits IL-1β	[101]	Inflammation and Cardiovascular risk	NCT02272946
Colchicine	Decreases IL-1β, IL-18, and IL-6	[100]	Peripheral artery disease	NCT04774159
			Perioperative AF	NCT03310125
Dapansutrile	Blocks NLRP3 assembly, Prevents NLRP3-induced release of IL-1β and IL-18	[103]	Schnitzler’s syndrome	NCT03595371
				NCT04540120
Inzomelid	Prevents ASC release and NLRP3 activation	[104]	Cryopryrin-Associated Periodic syndromes	EudraCT2020-000489-40
			Parkinson’s disease	NCT04338997
Pirfenidone	Decreases NLRP3 and ASC expression Inhibits caspase-1 activation and Prevents IL-1β maturation	[105]	Pulmonary Fibrosis	NCT03109288
				NCT03109288
SGLT2 inhibitors	Prevents IL-1β release	[107]	Myocardial Infarction	NCT04899479
Somalix	Inhibits NLRP3 activation	[104]	Peripheral arterial disease	NCT04015076
Tranilast	Binds NACHT domain of NLRP3 to block NLRP3 oligonerization	[106]	Mucinoses	NCT03490708
			Cryopryrin-Associated Periodic syndromes	NCT03923140

**Table 2:** Recent resolution strategies in experimental models of RHD. Review of recently tested resolution strategies in the treatment of arrhythmogenic structural and electrical remodeling resulting from severe pulmonary hypertension generated by MCT and SuHx in rats. RvD1 has been shown to significantly reduce atrial fibrous content while decreasing AF vulnerability in MCT rats. DA prevented ventricular arrhythmias via reduction of NFκB activity in MCT rats. RLX prevented atrial and ventricular fibrosis and fibrillation, while reducing the expression of notorious proinflammatory cytokines and proteins. Abbreviations: AF: Atrial Fibrillation; CHEMR23: chemerin receptor 23; COL: Collagenase; DA: dapaglifozin; ICAM1: intracellular adhesion molecule 1; IL: interleukin; i.p.: intra peritoneal; MMP: Matrix Metalloproteinase; MCT: monocrotaline; NLRP3: NACHT, LRR, and PYD domains-containing protein 3; NFκB: Nuclear Factor kappa B; NRF2: nuclear factor erythroid-derived 2; RLX: relaxin; RvD1: resolvin D1; sc.: subcutanous; SuHx: Sugen-Hypoxia; TGF-β: Transforming Growth Factor Beta; TLR4: toll-like receptor 4; VF: Ventricular Fibrillation.

Experimental Models of RHD	Resolution Strategies	Anti-Arrhythmogenic Effects	References
**Monocrotaline** **(MCT)**	Resolvin D1 (RvD1) (*i.p.: 2 µg/kg/d; 3w*)	• ↓ Atrial fibrosis • ↓ Expression of IL6, TGF-β, ICAM1, IL1β NLRP3-inflammasome • ↑ Expression of IL10, CHEMR23 • ↓ AF susceptibility	Hiram et al., 2021 [54]
	Dapagliflozin (DA) (*per os: 60 mg/L; 4w*)	• ↓ Ventricular fibrosis • Prevented channelopathy • ↓ TLR4 and NFκB activity • ↓ VF vulnerability	Qin et al., 2022 [127]
**Sugen-Hypoxia** **(SuHx)**	Relaxin (RLX) (*sc.: 30–400 µg/kg/d; 6w*)	• ↓ NRF2 and gluthathione transferase • ↓ Expression of TGF-β, MMP2, MMP9, COLI and COLIII • ↓ Ventricular fibrosis and VF • ↓ Atrial fibrosis and AF	Martin et al., 2021 [119] Parikh et al., 2013 [130]

## Abbreviations

AF	Atrial Fibrillation
CM	Cardiomyocytes
ECM	Extracellular Matrix
FB	Cardiac Fibroblasts
FRM	Failed Resolution Mechanisms
IL	Interleukin
MI	Myocardial Infarction
RA	Right Atrium
RHD	Right Heart Disease
SPMs	Specialized Pro-Resolving Mediators
